# CATALYZING CROSS-NATIONAL AGING RESEARCH: OPPORTUNITIES, CHALLENGES, AND STRATEGIES FOR EARLY CAREERISTS

**DOI:** 10.1093/geroni/igad104.1073

**Published:** 2023-12-21

**Authors:** Lindsay Kobayashi

**Affiliations:** University of Michigan School of Public Health, Ann Arbor, Michigan, United States

## Abstract

Cross-national comparisons in aging research allow for the triangulation of evidence on health outcomes across diverse populations and contexts. They allow researchers to identify policy-relevant and context-specific modifiers of health risks faced by older adults. Catalyzing cross-national comparison studies is a challenge, as researchers need to carefully consider social, cultural, political, socioeconomic differences across populations and make sure that local partners are appropriately represented and involved. This presentation will describe experiences in building a research career focused on understanding healthy cognitive aging from a global perspective, using data from diverse high-, middle-, and low-income settings. Opportunities, challenges, and strategies for early career researchers interested in catalyzing cross-national aging research will be discussed. These include the importance of advocacy for research on aging in contexts outside of the United States, theoretical and methodological approaches to cross-national research, and building diverse interdisciplinary collaborations.

